# Predicting malaria outbreaks from sea surface temperature variability up to 9 months ahead in Limpopo, South Africa, using machine learning

**DOI:** 10.3389/fpubh.2022.962377

**Published:** 2022-08-25

**Authors:** Patrick Martineau, Swadhin K. Behera, Masami Nonaka, Ratnam Jayanthi, Takayoshi Ikeda, Noboru Minakawa, Philip Kruger, Qavanisi E. Mabunda

**Affiliations:** ^1^Application Laboratory, VAiG, Japan Agency for Marine-Earth Science and Technology, Yokohama, Japan; ^2^Division of Natural Science Solutions, Blue Earth Security Co., Ltd., Tokyo, Japan; ^3^Department of Vector Ecology and Environment, Nagasaki University, Institute of Tropical Medicine, Nagasaki, Japan; ^4^Malaria Control Programme, Limpopo Department of Health, Tzaneen, South Africa

**Keywords:** malaria, prediction, machine learning, climate, weather, South Africa

## Abstract

Malaria is the cause of nearly half a million deaths worldwide each year, posing a great socioeconomic burden. Despite recent progress in understanding the influence of climate on malaria infection rates, climatic sources of predictability remain poorly understood and underexploited. Local weather variability alone provides predictive power at short lead times of 1–2 months, too short to adequately plan intervention measures. Here, we show that tropical climatic variability and associated sea surface temperature over the Pacific and Indian Oceans are valuable for predicting malaria in Limpopo, South Africa, up to three seasons ahead. Climatic precursors of malaria outbreaks are first identified *via* lag-regression analysis of climate data obtained from reanalysis and observational datasets with respect to the monthly malaria case count data provided from 1998–2020 by the Malaria Institute in Tzaneen, South Africa. Out of 11 sea surface temperature sectors analyzed, two regions, the Indian Ocean and western Pacific Ocean regions, emerge as the most robust precursors. The predictive value of these precursors is demonstrated by training a suite of machine-learning classification models to predict whether malaria case counts are above or below the median historical levels and assessing their skills in providing early warning predictions of malaria incidence with lead times ranging from 1 month to a year. Through the development of this prediction system, we find that past information about SST over the western Pacific Ocean offers impressive prediction skills (~80% accuracy) for up to three seasons (9 months) ahead. SST variability over the tropical Indian Ocean is also found to provide good skills up to two seasons (6 months) ahead. This outcome represents an extension of the effective prediction lead time by about one to two seasons compared to previous prediction systems that were more computationally costly compared to the machine learning techniques used in the current study. It also demonstrates the value of climatic information and the prediction framework developed herein for the early planning of interventions against malaria outbreaks.

## Introduction

Malaria is a major infectious disease affecting about 250 million people and claiming more than half a million lives every year (1). It is most prevalent in Africa where it accounts for about 95% of the cases worldwide. Sub-Saharan Africa is most strongly affected due to environmental conditions that are favorable for the spread of malaria by *Anopheles* mosquitos (2, 3), the primary vector of the *Plasmodium falciparum* parasites causing the disease.

Local weather observations such as rainfall and temperature have been traditionally used to infer the number of expected malaria cases (4–23). This inference is based on the influence of weather on the life cycles of both the vectors and parasites responsible for malaria transmission. Specifically, rainfall provides good conditions for breeding sites thus exerting an important control on mosquito reproduction (12, 16, 24, 25). It generally takes about 6 to 8 weeks for a malaria outbreak, a surge in malaria cases above typical levels, to occur from the time of a rainfall event given the life cycle of the mosquito and the infection cycles of the parasite in the mosquito and human hosts. In addition to precipitation, the temperature is also known to influence the survival and development of *Anopheles* mosquitos (26–28), and incubation of *Plasmodium falciparum* (29–31).

Over the years, various attempts were made to develop early warning prediction systems (32) based on those local weather observations, either process-based (33, 34) or statistical-relationship-based (25, 35–38). Such predictions and traditional knowledge based on empirical relationships have been used by malaria control centers for local malaria predictions, but the time separating malaria outbreaks from rainfall events is usually too short, especially in under-developed and unindustrialized nations, to procure the additional budget and resources necessary for an intervention strategy, such as providing mosquito nets and spraying insecticides. In addition, it is required for efficient planning of interventions to predict malaria outbreaks one to two seasons ahead, but it is not possible to predict local weather conditions that far in time (39). Ensemble coupled ocean-atmosphere climate predictions, which exploit the predictive power of coupled modes of climate variability, were shown to be effective in extending the prediction range (40, 41) but only by a few weeks besides being computationally heavy.

Modes of climate variability that are of large spatial scale and slowly evolving, such as El Niño–Southern Oscillation (42) (ENSO) and the Indian Ocean Dipole (43) (IOD), which affect the evolution of tropical sea surface temperatures (SSTs), offer a potential source of predictability at long lead times. These modes result from the coupling between the ocean and the overlying atmosphere, with the former providing the memory that is a source of skill in long-term predictions (44, 45). Tropical modes of variability, through their influence on tropical precipitation and resultant teleconnections to the extratropics (46), can have broad impacts on temperature and precipitation, such as the influence of ENSO and IOD on African climate. More specifically, below-normal precipitation is usually expected in Eastern South Africa during El Niño (47–49) through weakened moisture flow. The IOD was demonstrated to have a comparatively more important impact on East-African short rain events through a modulation of moisture transport by the Walker circulation (50). In fact, ENSO (8, 51, 52) and IOD (14, 53, 54) have been linked with malaria incidence in South Africa. In addition to tropical variability, SST variability in the subtropics associated with the Indian Ocean Subtropical Dipole (IOSD) (52), and SST variability in the southwestern Indian Ocean (55) have also been identified as precursors of malaria in Africa. SST variability has also been proven useful in predicting malaria outbreaks over other sectors such as India (56) and cholera outbreaks (57).

The present study seeks to identify and make use of those untapped large-scale climatic precursors of malaria outbreaks using an extensive but cost-effective suite of machine-learning techniques designed to provide early malaria warnings with lead times up to two to three seasons with high accuracies. We aim to provide predictions for seasonal averages since weather factors that may lead to variations in malaria incidence at shorter time scales (days to weeks) are not predictable at the long prediction lead times considered (39). The prediction framework developed herein aims to predict malaria cases in the South African province of Limpopo—for which malaria statistics are consistently archived since 1998—as a proof of concept of the feasibility of long-range malaria predictions based on climate variability.

## Methods

### Data

#### Malaria data

**Figure 1:**
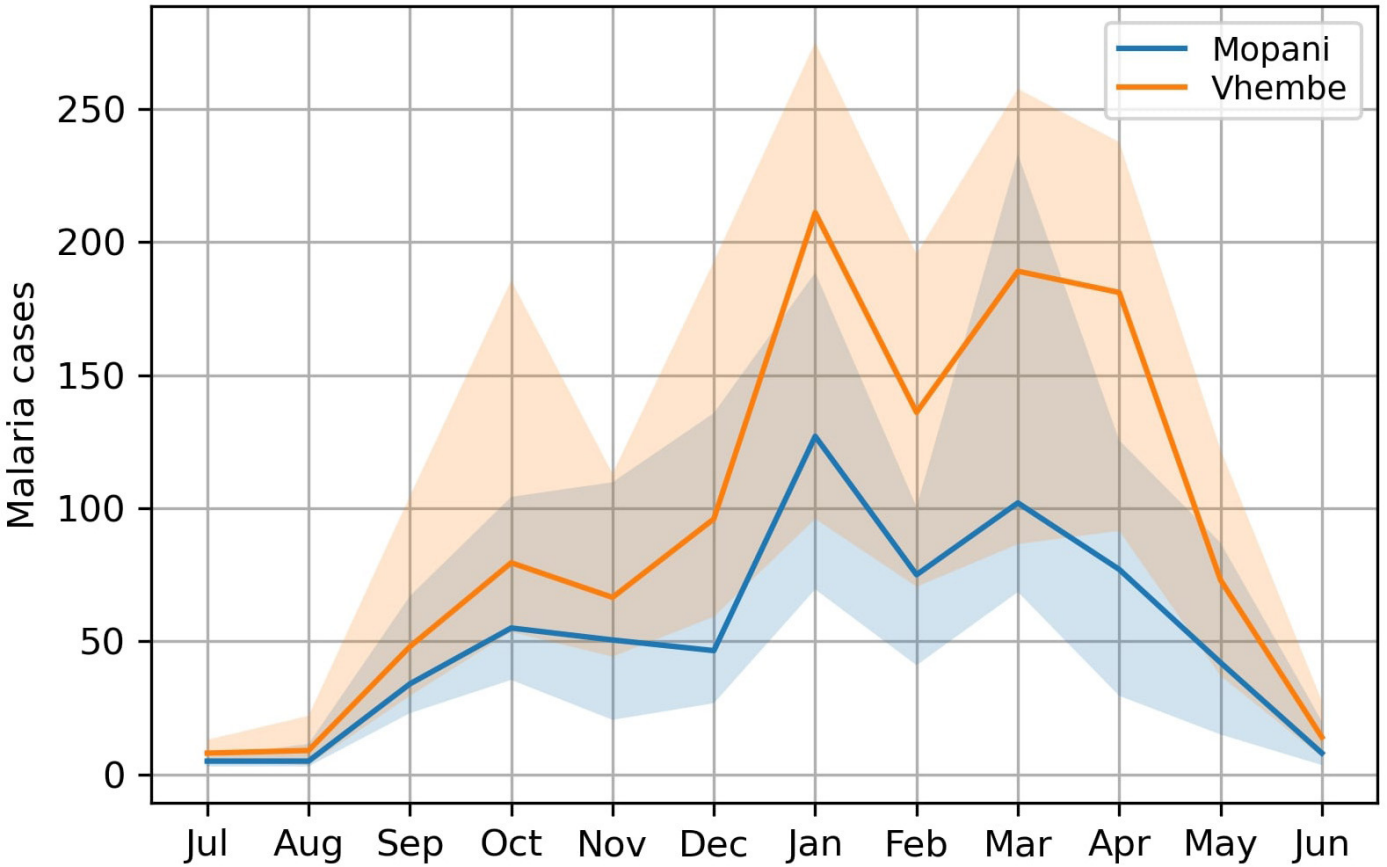
Seasonal cycle of malaria incidence in South Africa. The seasonal cycle of monthly malaria incidence (median) is illustrated for the districts of Vhembe and Mopani, where the highest malaria incidences are reported in the province of Limpopo, South Africa. The highest incidences are observed in austral summer when temperature and precipitation are favorable for the spread of malaria. Interannual variations as measured with the interquartile range (25th percentile to 75th percentile) are shaded.

The monthly malaria case count is compiled from 1998 to 2020 based on data (58) provided by the Malaria Institute in Tzaneen, South Africa. This period corresponds to the availability of data at the beginning of the development of our machine-learning models. South African data is used here for its quality and long-term consistency. This work focuses on malaria outbreaks affecting the district of Vhembe situated in South Africa's northernmost province, Limpopo. This district experiences some of the most severe malaria outbreaks of all South Africa. Results for the district of Mopani, which experiences the second-largest outbreaks, are briefly explored. For this study, only passively surveyed cases (patients seeking medical care) from hospitals and health centers are compiled, avoiding the double-counting of patients consulting both at private clinics and larger health centers. The analysis performed in our study was carried out separately for each district but for brevity, only the results for the Vhembe and Mopani districts, where malaria cases are highest in Limpopo, are presented in this work. In both districts, malaria incidence is maximized and subject to the largest interannual variations in late austral summer (Figure 1). A broad maximum comprises two local maxima found before and after the month of February and a minor peak is observed in October. Bimodal malaria patterns were observed in other countries (59) and associated with the long and short rainy seasons. We note however that rainfall shows a single January peak in Limpopo (52) and that bimodal distributions with well-separated double peaks are usually found closer to equatorial Eastern Africa (50).

Malaria data, unless otherwise stated, is subjected to a 3-month smoothing window. Thus, the predictions presented herein are for entire seasons (e.g., December-January-February, January-February-March, etc.). For brevity, predictions (or dependent variables) are labeled with their central month (January for a December-January-February prediction). It is not the goal here to predict outbreaks with a precision of days or weeks. Weather factors that may lead to variations in malaria incidence at such a short time scale are not predictable at the long prediction lead times considered (43). As discussed later, using unsmoothed monthly data reduces the prediction accuracy demonstrating that consideration of longer time scales of variability is required for accurate early warning predictions with lead times of seasons.

#### Climate data

Gridded (latitude by longitude) monthly time series of climate variables (Table 1) are obtained from the NCEP/NCAR reanalysis (60), the CPC Merged Analysis of Precipitation (CMAP) (61), and the NOAA OISST (62) datasets. All these datasets are regularly updated to include the most recent observations.

**Table 1:** List of climate variables with their origin, units, and spatial resolution.

**Dataset**	**Variable**	**Label**	**Units**	**Resolution**
OISST: Optimum Interpolation Sea Surface Temperature	sea surface temperature	sst	°C	1° by 1°
CMAP: CPC Merged Analysis of Precipitation	precipitation	precip	mm/day	2.5° by 2.5°
NCEP/NCAR Reanalysis 1	surface pressure	pres	Pa	1.875° by 1.9°
	2 m mininum temperature	tmin	K	1.875° by 1.9°
	2 m maximum temperature	tmax	K	1.875° by 1.9°
	10 m eastward wind	uwind	m/s	1.875° by 1.9°
	10 m northward wind	vwind	m/s	1.875° by 1.9°

### Lag-regression analysis

To identify SST precursors of malaria, we perform a lag-regression analysis where SST is linearly regressed onto malaria incidence according to the model function *V*(*t*+τ) = *a*+*bM*(*t*), where *V* is a monthly SST time series, *M* is the monthly malaria count, *t* is time, and τ is lag. Malaria count is chosen as an explanatory factor so that maps of the coefficients *b* illustrate the typical SST anomalies associated with malaria outbreaks. All data are first detrended before assessing the regression. The calculation is performed grid-by-grid to obtain maps of correlation coefficients *r*(τ, ϕ, λ) that are a function of lag *(*τ*)*, latitude (ϕ), and longitude (λ). Correlation coefficients are assessed for lags ranging from 0 months (instantaneous) to −12 months. To identify seasonally varying precursors, this analysis is carried out separately for each month of the year. For instance, *r*_*february*_(τ = −1, ϕ, λ) is obtained by regressing *V* in January onto *M* in February, thus describing the typical state of V observed one month before malaria fluctuations occurring in February. A similar analysis is carried out for the local climate indices described in the next section. For the lag-regression analysis, malaria and climate data are subjected to a 3-month running mean.

**Figure 2:**
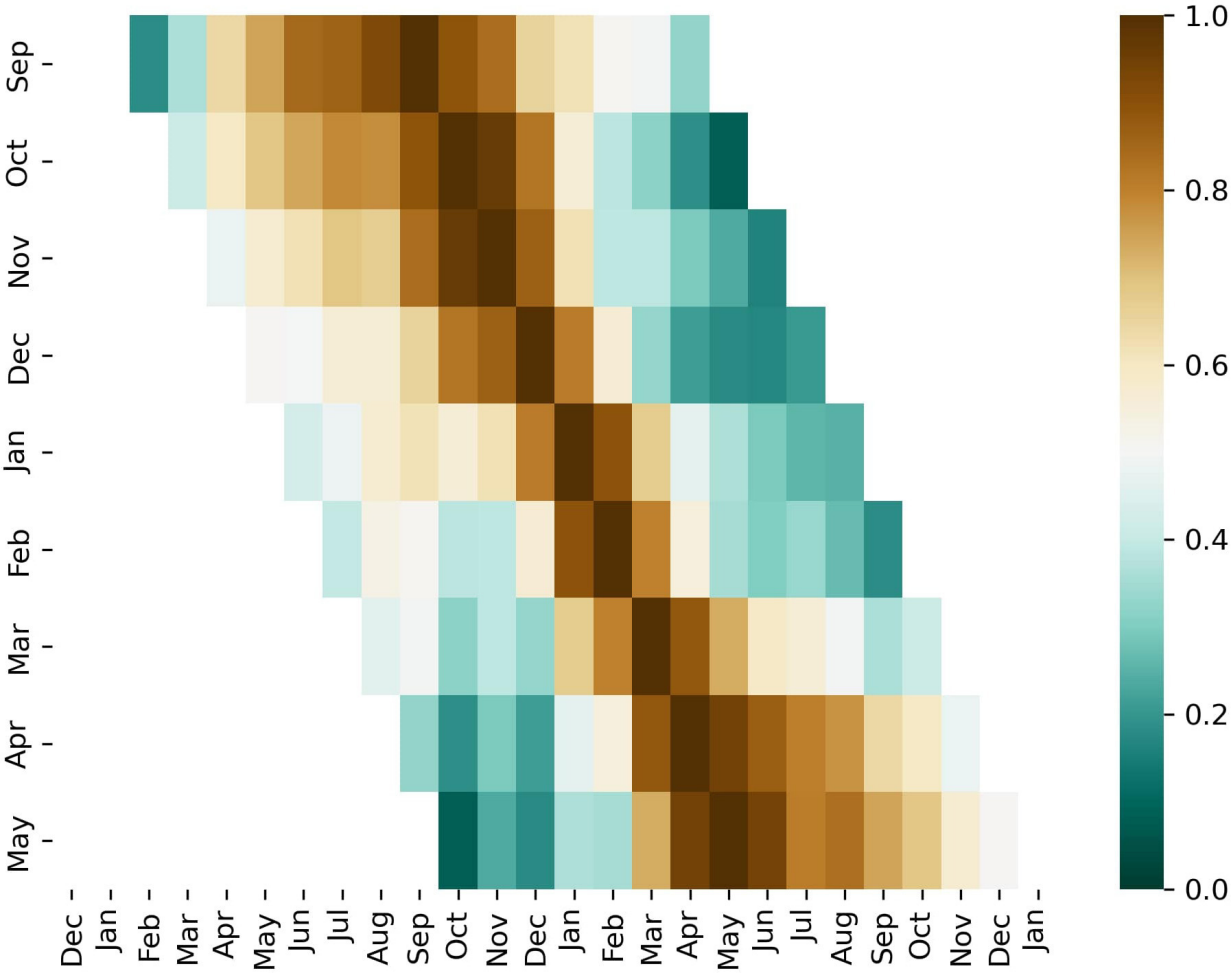
Persistence of malaria incidence. A cross-correlation of malaria incidence for various reference months. Correlation is indicated in color. For a specific month (y-axis) the correlation of malaria incidence with respect to other months are shown along the x-axis.

We note that malaria incidence in September and October is weakly correlated with the incidence in April and May indicating a weak persistence throughout the high-risk season (Figure 2). We also note that early outbreaks (July, September, October, November) and late outbreaks (March, April, May) tend to be better correlated (slow decrease of correlation further away from the reference month) while the outbreaks occurring in January and February tend to be less correlated with other months. These findings overall indicate a break in the persistence of malaria incidences occurring between the early and late seasons perhaps due to different climate precursors, as confirmed by our analysis, and hence forecasts for the beginning and end of the high-risk season should be performed independently. We note that the break in persistence cannot be explained by a bimodality of rainfall distribution as rainfall seasonality exhibits a single January peak over Limpopo (52).

### Climate indices

**Figure 3:**
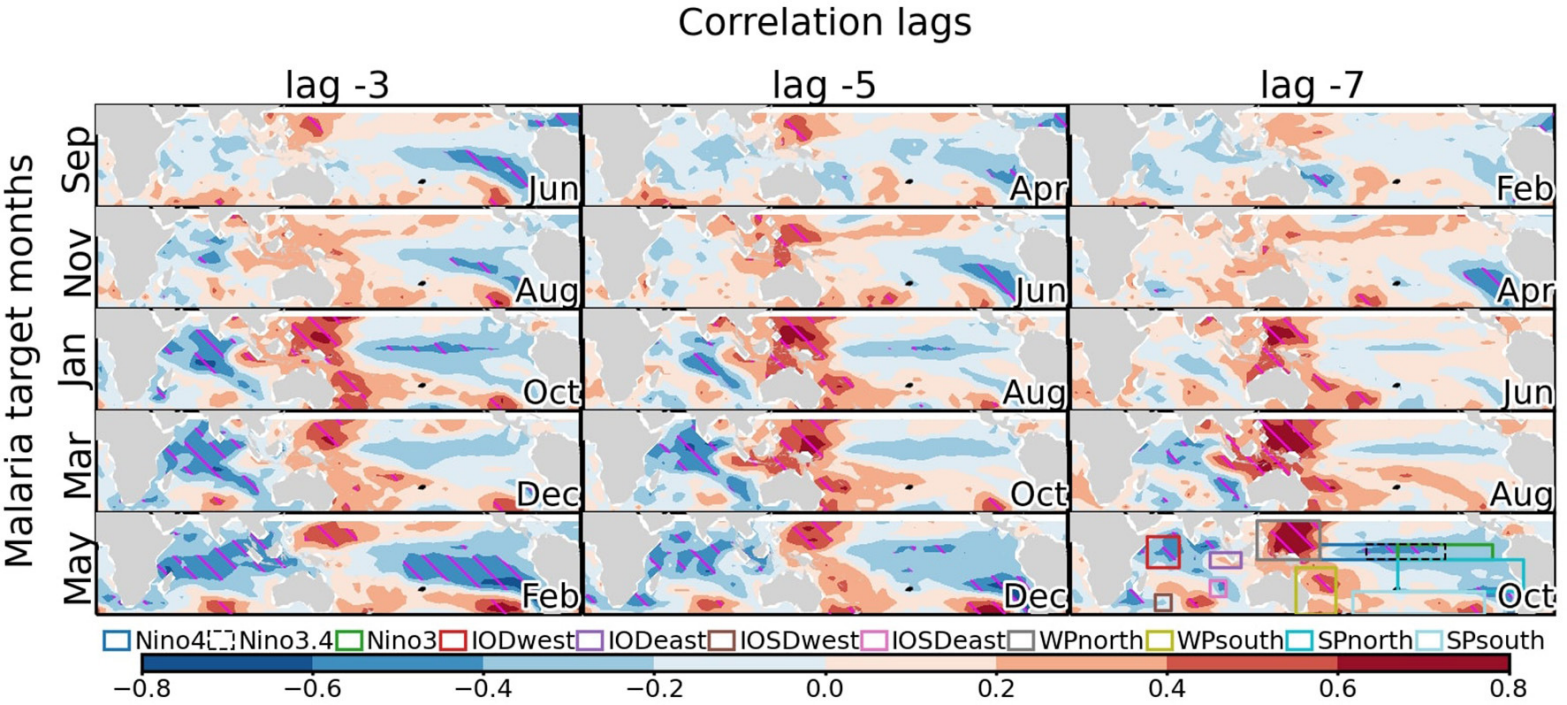
Sea surface temperature precursors of malaria in South Africa. Lag-regression maps of SST on malaria incidence in South Africa (Vhembe district in Limpopo province) are shown in function of target malaria month (rows) and lag (columns). The month when the precursors are observed is indicated in the bottom-right corner of each panel. For example, lag −3 precursors of observed malaria incidence in January are observed in October. Correlation is shaded at an interval of 0.2 with blue and red shadings for negative and positive correlations, respectively. Correlations that are significant at the 5% significance level are hatched in magenta. The conditions illustrated are associated with high malaria incidence. See Supplementary Figure 1 for the location of Limpopo. The sectors used later to construct SST-based climate indices are illustrated in the bottom-right panel according to the legend.

Based on the analysis of precursors performed in this study and previous studies on the connection between malaria and climate variability (8, 51–55, 63), we investigate the usefulness of tropical and subtropical modes of SST variability for predicting malaria. The domains used to compute the indices characterizing these modes are listed in Table 2 and illustrated in Figure 3 as well as in Supplementary Figure 1. Local climate indices that are based on precip, tmax, tmin, pres, uwind, and vwind are also considered. They are obtained by averaging these variables over the South African province of Limpopo and Mozambique (Supplementary Figure 1).

**Table 2:** Local and global climate predictors used to predict malaria incidence and their domains.

**Scope**	**Variable/Index**	**Sector**	**Latitude range**	**Longitude range**
**Local**	precip	LP/MZ	—	—
	tmax	LP/MZ	—	—
	tmin	LP/MZ	—	—
	pres	LP/MZ	—	—
	uwind	LP/MZ	—	—
	vwind	LP/MZ	—	—
**Global (SST)**	IODeast	Indian Ocean	10°S−0°S	90°E−110°E
	IODwest	Indian Ocean	10°S−10°S	50°E−70°E
	IOSDeast	Indian Ocean	28°S−18°S	90°E−100°E
	IOSDwest	Indian Ocean	37°S−27°S	55°E−65°E
	Niño3	Pacific Ocean	5°S−5°N	210°E−270°E
	Niño3.4	Pacific Ocean	5°S−5°N	190°E−240°E
	Niño4	Pacific Ocean	5°S−5°N	160°E−210°E
	WPsouth	Pacific Ocean	40°S−10°S	145°E−170°E
	WPnorth	Pacific Ocean	5°S−20°N	120°E−160°E
	SPsouth	Pacific Ocean	40°S−25°S	180°W−95°W
	SPnorth	Pacific Ocean	25°S−5°S	150°W−70°W

Local climate variables are averaged over Limpopo (LP) and Mozambique (MZ). The coordinates delimiting the sectors where SST is averaged for the indices representing variability over the tropical Pacific Ocean associated with ENSO (Niño3, Niño3.4, and Niño4), the Indian Ocean Dipole mode (IODeast and IODwest), the Indian Ocean Subtropical Dipole (IOSDeast and IOSDwest), western subtropical Pacific variability (WPnorth, WPsouth), and South Pacific variability (SPnorth, SPsouth) are indicated. These sectors are illustrated in Figure 3 and Supplementary Figure 1. The IOD index is obtained as IODwest-IODeast and the IOSD index as IOSDwest-IOSDeast.

SST anomalies outside the tropics are also observed to be associated with malaria incidences (not shown), but these are typically considered to be driven by atmospheric variability (64) on the seasonal time scale considered in this work and may even be driven by tropical SST variability through atmospheric teleconnections (65). They are thus not investigated.

The SST variability considered herein includes variability associated with the Indian Ocean Dipole (IODeast and IODwest in Figure 3 and Supplementary Figure 1), a prominent seesaw of temperature variability in the tropical Indian Ocean (43), the Indian Ocean Subtropical Dipole (IOSDeast and IOSDwest) characterized by dipolar anomalies in the southern Indian Ocean (66), the El Niño–Southern Oscillation (ENSO; characterized by Niño indices) which strongly affects eastern Pacific SST anomalies (42). In addition to these well-recognized modes of SST variability, we add sectors to characterize SST variability in the western Pacific (WPsouth and WPnorth) and in the southern Pacific (SPsouth and SPnorth).

We note that these climate predictors are not necessarily independent from each other. Cross-correlations are often observed (e.g., Supplementary Figure 2). For instance, WP indices are anticorrelated with Niño indices suggesting that the earlier are associated in part with ENSO variability (67).

The time scale of variability of the resulting climate indices is illustrated *via* their autocorrelation in Supplementary Figure 3. We note that SST-based climate indices tend to be markedly more persistent in contrast to local indices.

### Machine-learning techniques

The task of predicting malaria outbreaks is here set up as a classification problem where for each month of the year, we attempt to predict whether the malaria case count is higher or lower than usual as independently defined for each calendar month's historical median value to account for the seasonal cycle of malaria (Figure 1). Thus, our predictions do not depend on the seasonal cycle. It is predicted whether the malaria cases are higher or lower than usual relative to each month's historical incidences. A set of machine-learning classifiers is then trained to predict the occurrence of each category using the defined climate indices as predictors. Because the climatic precursors are herein demonstrated to be seasonally varying, training and prediction are performed month-by-month, which removes any seasonality in the data. To identify sources of predictability, a series of experiments are carried out with various subsets of predictors (Table 3).

**Table 3:** List of experimental setups.

**exp#**	**Malaria predictors**	**Climate predictors**
1	•	SST_[IODwest,IODeast]_
2	•	SST_IODwest_
3	•	SST_IODeast_
4	•	SST_IOD_
5	•	SST_[IOSDwest,IOSDeast]_
6	•	SST_IOSDwest_
7	•	SST_IOSDeast_
8	•	SST_IOSD_
9	•	SST_NINO[3,3.4,4]_
10	•	SST_WP_
11	•	SST_SP_
12	•	SST
13	•	precip, tmax, tmin
14	•	pres, vwind, uwind
15	•	local
16	•	all
17	•	-
18		SST_[IODwest,IODeast]_

The details of each experimental setup are indicated. A complete list of climate predictors is provided in Table 2. “all” denotes all of the climate indices listed in the table.

To perform the predictions, a set of classifiers (Supplementary Table 1) are trained to predict the aforementioned malaria incidence classes using the climate indices defined in the previous section. Training is performed independently for each calendar month and lead time to account for the seasonally-varying precursors. It would be possible to train the classifiers with all months of the year simultaneously if using non-linear classifiers such as random forests, but the inclusion of linear classifiers in our study prevents such an approach. All the classifiers are provided in the scikit-learn (68) Python module. With these classifiers, predictions are prepared for lead times ranging from 1 to 12 months, in steps of 1 month (Supplementary Figure 4). As a standard, predictors are provided only for the last month of observations. We note that whereas general statistical relationships were investigated with linear assumptions in the previous section, some of the classifiers used herein can resolve more complex non-linear climatic influences.

For model training and evaluation, we use nested cross-validation (Supplementary Figure 5). This method consists of two loops where the inner loop is nested into the outer loop. The inner loop is used to carry out hyperparameter tuning of each classifier. These hyperparameters describe the characteristics of the classifiers and cannot be trained. Predictions using many combinations of hyperparameters are performed and the best parameters, based on accuracy, are retained for future predictions. Precise identification of hyperparameters is out of the scope of this work. The best hyperparameters are selected based on the prediction accuracy of validation data. It is also with the accuracy to predict validation data that the models with the best accuracies (three-best and five-best) are chosen to construct multi-model voting ensembles (Supplementary Table 1). We note that, unlike climate model ensemble members which use the same models with different initial conditions, our voting ensemble combines the votes of different classifiers. The models that are included in these ensembles are selected separately for each calendar month and prediction lead time. Since the multi-model voting ensembles are constructed based on the outcome of the inner loop (without knowledge of the test data; see Supplementary Figure 5), its accuracy as evaluated in the outer loop adequately reflects the expected operational accuracy. For the inner loop, we use a leave-one-out approach to select validation data.

Once hyperparameter tuning and model selection for the multi-model voting ensembles have been carried out in the inner loop, all models are retrained with the entire samples of the inner loop (including the validation data set aside). Then, in the outer loop, test data are put aside each iteration to assess the overall accuracy of predictions resulting from the process of hyperparameter tuning and ensemble model selection. Here we use a leave-two-out technique where two samples (2 months) are put aside as test data for all possible combinations and n-2 samples are used for training, where n is the total sample size. This validation technique is superior for small sample sizes, as in the case of malaria predictions since it allows training on a maximal amount of data and thoroughly assesses accuracy with all samples available. For each of these iterations of the outer loop, iterations over the inner loop are performed.

### Smoothing

Climate indices are susceptible to being affected by high-frequency weather noise which may adversely affect model training and predictions. This is especially true for the local indices constructed over Limpopo and Mozambique since their areas are relatively small (see Supplementary Figure 1) and thus affected by small-scale transient weather systems. Similarly, noise in malaria data may also be detrimental to predictions. As a standard, we apply a 3-month running mean to both climate indices and malaria data.

In experiments using a 3-month smoothing window for malaria data, we labeled malaria predictors using the last month used. For example, malaria incidence labeled as March 2020 is an average of malaria cases in January, February, and March. We note that with such convention, there is a 1-month overlap between the averaging windows of malaria predictors and malaria predictands at a 1-month lead which may artificially inflate accuracies. We note however that this is not an issue here as such a short lead time is not the focus of our work. Similarly, climate predictors (or independent variables) are labeled using the last month used in the smoothing window to emulate real operational capabilities.

### Control experiment and sensitivity experiments

To understand the impact of various modeling techniques used in our malaria prediction framework on prediction accuracy, we perform various experiments where predictors (Table 3) and prediction techniques (Supplementary Table 2) are altered. The various techniques that are switched on or off are described below. As a basis for comparison, the standard prediction setup for which we obtained overall the highest prediction accuracies, described in more detail in this manuscript, is referred to as the control experiment (exp1). It uses malaria case data and SST-based climate indices over the tropical Indian Ocean (IODwest and IODeast).

#### Malaria predictors

If historical malaria data are provided, they are provided only for the most recent month of predictor history (Supplementary Figure 4). It is provided as a normalized quantity, thus giving a sense of whether it is higher or lower than usual for the specific month it is provided. To test whether malaria predictors provide predictive power, they are excluded in one experiment (exp18) and provided as the sole predictor in another (exp17; Figure 5).

#### Climate predictors

To understand the value of climate predictors, we perform experiments where predictors are limited to various combinations of SST-based indices (exp1-12) and local variables (exp12-15). SST-based indices are also further divided whether they describe SST variability associated with the Indian Ocean Dipole (IOD; exp1-3), the Indian Ocean Subtropical dipole (IOSD; exp5-8), ENSO (NINO; exp9), the SST precursors identified over the western Pacific sector (WP; exp10; see Figure 3), and subtropical South Pacific variability (SP; exp 11). The domains used to construct these indices are indicated in Figure 3, Supplementary Figure 1 and Table 2.

### Assessment of skill

Since the classes of malaria predicted in this study are balanced, we can use the accuracy, defined as the ratio of successful predictions to the total number of predictions, to characterize the skill of our predictions without worrying about unbalances in the representation of classes:


$$\begin{array}{l} \text{Accuracy}=\frac{\#\text{Success}}{\#\text{predictions}}=\frac{{\text{T}}_{\text{N}}\;+\;{\text{T}}_{\text{P}}}{\text{P }+\text{ N}} \end{array}$$

where T_N_ and T_P_ are the number of true negatives and true positives, respectively. Qualitatively similar results are obtained by analyzing the sensitivity, specificity, F1 score (not shown), and area under the receiver operating characteristic (ROC) curves (69) (Supplementary Figure 6). Only the accuracy is reported in the main results for brevity.

### A note on below-chance accuracies

Prediction accuracies and the area under the curve below chance levels (<0.5) are sometimes observed (e.g., Figure 5), suggestive of anti-learning (70). Flipping these predictions would provide above-chance accuracies if the decision to flip them could be made based on the training data, without knowledge of the prediction accuracy of test data (see Supplementary Figure 5), as would be the case for a real prediction. It is however not possible here since we observe that in these instances, the prediction accuracy of the training data is well above 0.5 (not shown), despite the below-chance accuracy of test data. In other words, not until the outcome of the prediction is known are we aware of the below-chance performance, thus no action can be taken ahead of time. Below-chance accuracies were shown to occur for small sample sizes when the effect of a predictor on the predictand is too small to be detected by a classifier (70–72). Below-chance accuracy should thus be interpreted here as an absence of prediction skill.

### Limitations on prediction accuracy

One limitation of the prediction method developed herein is the exclusion of socioeconomic factors, which also influence infection rates (73, 74). For instance, cross-border migration can contribute to up to 70% of observed cases in South Africa (75), thus year-to-year changes in migration may influence malaria infection rates. It cannot however be included in the machine-learning techniques developed herein because it is not adequately monitored (75, 76). The sharing of malaria case data together with human migration data among South African counties would be helpful to further improve such early warning systems of malaria. Other potential socioeconomic factors include temporal variations in the malaria control efforts, which have an important impact on malaria incidences but are not sufficiently documented to be included here as predictors. It may thus be best practice to consider that the predictions provided are produced for typical socioeconomic conditions and to carefully consider changes in social factors when developing planning interventions.

In addition, interdecadal changes in the coupling between modes of SST variability and their teleconnection to South African weather as well as noise in the reporting of malaria case data can introduce uncertainties which reduce prediction accuracy.

## Results

### Climatic precursors of malaria

Potential predictors of malaria outbreaks are first identified through lag regression analysis. Given the documented influence of modes of SST variability on the South African climate and malaria incidences, we first investigate their statistical relationship in more detail (Figure 3). A striking feature is the seasonality of these precursors throughout the malaria season (see Figure 1 for the seasonal cycle of malaria in Vhembe, Limpopo). This seasonality could be partly attributed to seasonal modulations of the extratropical response of tropical modes of variability (46) and/or the seasonal phase-locking of ENSO and IOD variability (43, 77, 78). Some of the most important precursors at a lag of −3 months (one season) include cold anomalies over the Indian Ocean (preceding malaria outbreaks in January-May), warm anomalies in the western Pacific located east of Australia and the Philippines (preceding malaria outbreaks in November-May), and cold anomalies over the eastern equatorial Pacific associated with La Niña (preceding malaria outbreaks in September). We also note a dipole in the subtropical south Pacific preceding outbreaks in May. SST precursors of malaria outbreaks also depend on the time separating the precursors from the occurrence of malaria outbreaks, which is likely a consequence of the periodic variability of the modes of tropical SST variability and their lagged influences on the South African climate. This dependence is most obvious for the precursors of malaria outbreaks in May over the eastern Pacific, which shows amplified cold anomalies at shorter lags.

**Figure 4:**
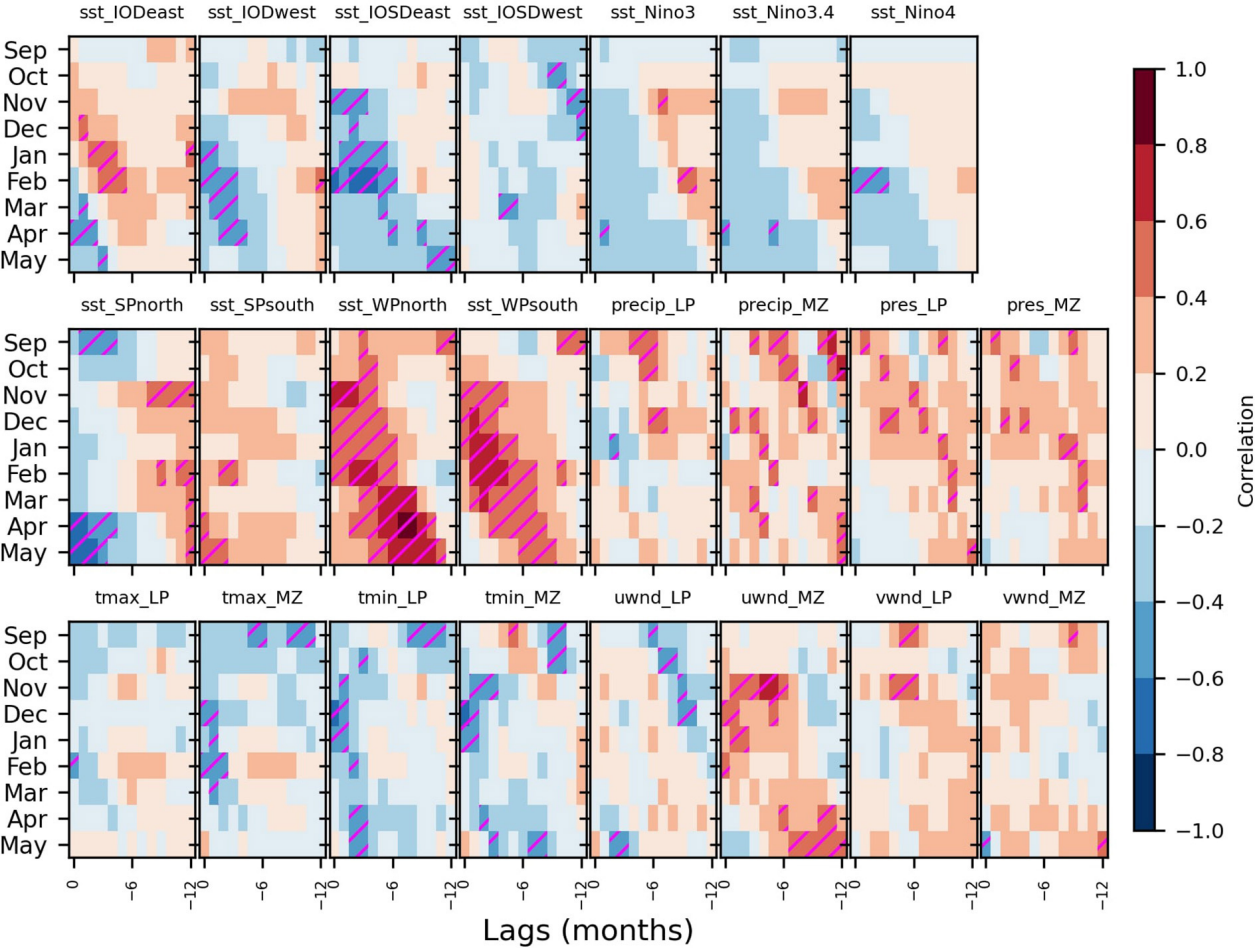
The lagged relationship between climate indices and malaria incidence in South Africa. Correlations between climate indices and malaria incidences throughout the malaria season (y-axes) and lags (x-axes) are illustrated with color shadings. Correlations that are significant at the 5% significance level are hatched in magenta. They are reported for various climate indices indicated above each panel (listed in Supplementary Table 1). The sectors used for SST indices (IODwest, IODeast, IOSDwest, IOSDeast, Niño3, Niño3.4, Niño4, SPnorth, SPsouth, WPnorth, and WPsouth) and local indices (precip, pres, tmax, tmin, uwnd, and vwnd; with the subscripts LP and MZ if averaged over Limpopo or Mozambique, respectively) are illustrated in Supplementary Figure 1. Domain boundaries are indicated in Table 2.

To investigate further the lagged relationship between SST precursors and malaria outbreaks we then examine in more detail documented modes of tropical SST variability. To this end, we select modes that are known to have a remote impact outside the tropics and we also consider new indices defined here to capture SST variability in the western Pacific (WP) and over the subtropical south Pacific (SP; Figure 3, Table 2, and Supplementary Figure 1). As noted before, the lagged relationship between malaria incidence and these modes of SST variability varies with respect to target malaria season and lag time (Figure 4). In austral spring, cold precursors are found primarily over the IOSDwest sector. In austral summer warm precursors include IODeast, WPnorth, and WPsouth and cold SST anomalies are found over the IODwest and IOSDeast sectors. In comparison, cold Pacific precursors (52, 55), as captured by the Niño indices, are less robust in our analyses. Although regression maps reveal prominent La-Niña-like cold anomalies in the eastern tropical Pacific (not shown), the correlation with malaria incidence is not as large as with other sectors. Finally, in austral fall, cold precursors are observed over SPnorth and warm precursors are seen for the SPsouth, WPnorth, and WPsouth sectors. We note that SST variability over the WP sectors is partly related to ENSO variability as the WP and ENSO indices are significantly anticorrelated (e.g., Supplementary Figure 2).

Atmospheric variables (surface temperature and precipitation), that are expected to have a direct impact on the life cycles of the *Anopheles mosquito* and *Plasmodium falciparum parasite*, and other variables indicative of the passage of weather systems (surface pressure and surface wind) affecting those atmospheric variables are analyzed over Limpopo. Since temperature and precipitation over Mozambique were also shown to be associated with malaria outbreaks in Limpopo (52), likely through importation of cases originating from Mozambique *via* cross-border human migration (75), we also consider their variability here. Among the important precursors, there is an overall tendency for precipitation to be enhanced over a period of 12 months leading to malaria outbreaks in the early malaria season (Sep-Dec) of Limpopo and Mozambique. Surface pressure also generally shows positive correlations, albeit weaker. Minimum temperatures are usually colder than usual at long lags before austral spring malaria outbreaks. Zonal wind (uwnd) over Mozambique also shows a strong linear relationship with malaria outbreaks with large positive correlations in the few months leading to malaria outbreaks in austral spring and summer. Enhanced meridional winds (vwnd) over Limpopo typically precede malaria outbreaks occurring in austral spring and summer.

It is found that local and global indices sometimes share an important fraction of variance (Supplementary Figure 2), indicating that local variables are under the influence of climate modes of variability through atmospheric teleconnections, which contribute to their variability on slower time scales. In this way, the tropical modes of SST variability could influence malaria in Africa through the modulation of local weather conditions. Hence, the predictive power of these climate indices, slowly varying with longer prediction lead time, is examined in the following section.

### Predicting malaria with machine learning

**Figure 5:**
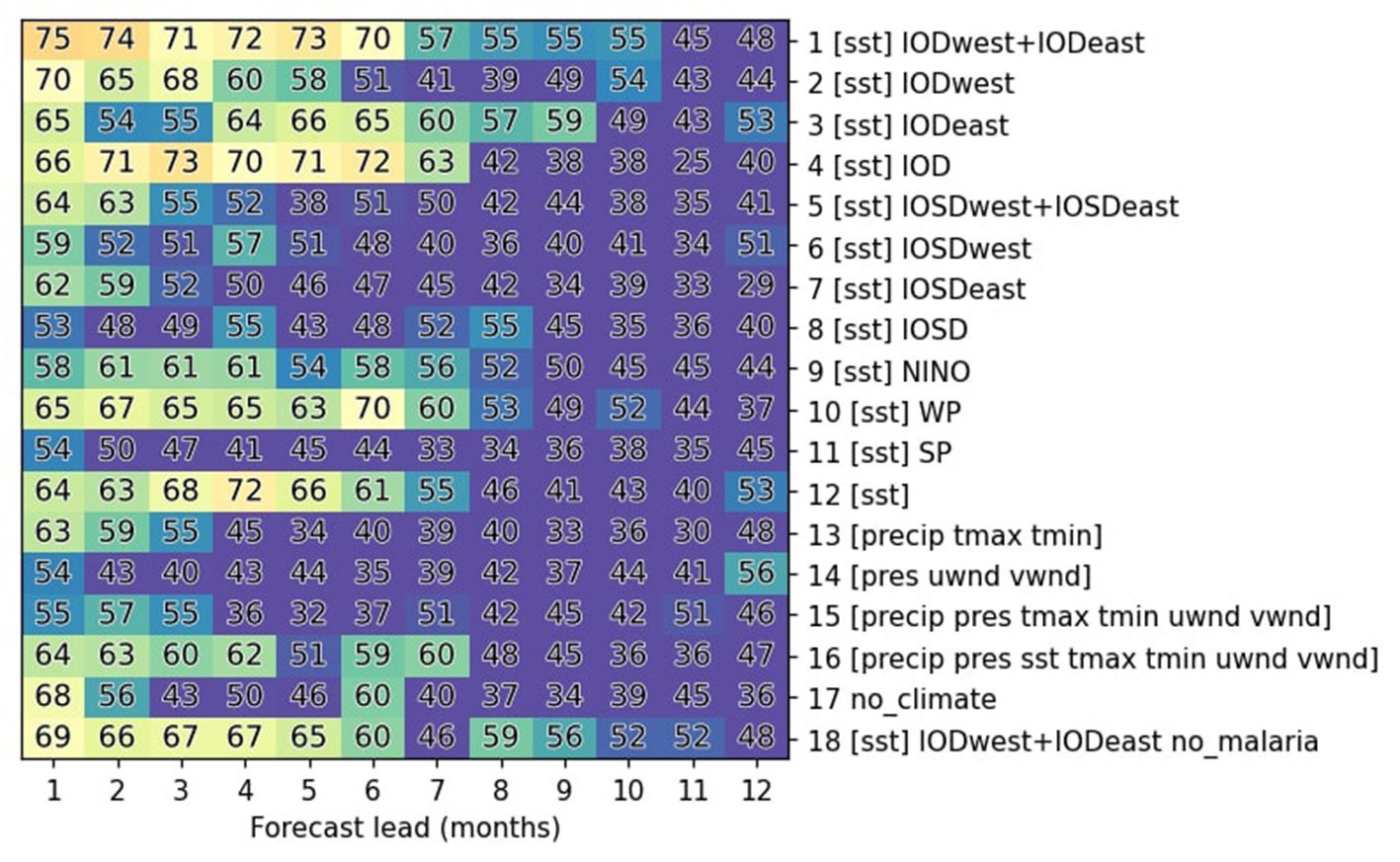
Summary of malaria prediction skills for various experimental setups. Malaria prediction accuracy is averaged over the months of December-April when malaria incidence is high (Figure 1). It is reported for a multi-model voting ensemble composed of the three best classifiers for each experimental setting (y-axis) and prediction lead (x-axis). Accuracy is indicated with numbers as the percentage of successful predictions and illustrated with colors with warmer colors indicate higher accuracies. Accuracies below 50 % are shown in purple.

To predict malaria, the climate indices investigated in the previous section and malaria case data are provided as predictors and predictand (dependent variable), respectively, for the training of machine-learning models that are described more extensively in the methods section. Various configurations of prediction techniques and selections of predictors are investigated to optimize model performance (Table 3, Supplementary Table 2). It is found that for December-April, some of the most accurate predictions are obtained by providing only SST-based climate indices over the tropical Indian Ocean (exp1; IODwest and IODeast) along with past malaria cases, with accuracies above 70% up to a lead time of 6 months (Figure 5). This accuracy is better compared to those obtained with only IODwest (exp2) or IODeast (exp3). Predictions based on the IOD index, calculated as their difference (IODwest-IODeast; exp4), has slightly reduced accuracy at shorter leads but with slightly better accuracies at longer leads compared to exp1. It is also worth noting that the accuracy is slightly lower when providing all SST indices (exp12) compared to only IOD-related indices (exp1). This likely is indicative of the overfitting of training data by the classifiers. In contrast, providing information about local weather/climate variables results in a much lower prediction skill on long lead times (exp13, exp14, and exp15), and providing all variables considered in this study (exp16) yields a reduced accuracy compared to providing only IOD-related indices (exp1), again likely because of overfitting. An important drop in accuracy is observed at long leads when attempting to predict malaria purely based on previous malaria incidences without climate indices (exp17), clearly showing impact of climate modes. Meanwhile, knowledge about the past evolution of malaria is nonetheless important, as the prediction accuracy decreases when it is excluded from predictions (comparing exp18 to exp1). Interestingly, except for the predictions based on SST variability over the western Pacific (exp10), which displays more moderate skill, other SST-based indices are of little value for long-term predictions for this period of the year.

**Figure 6:**
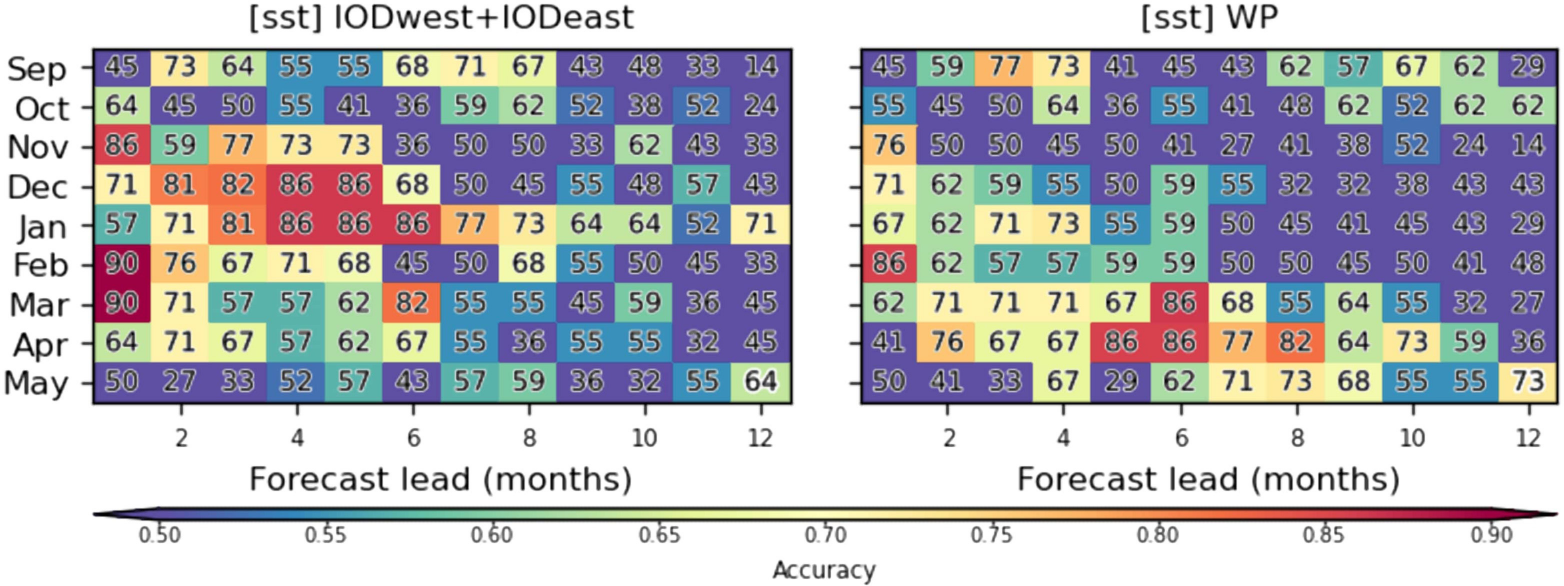
Seasonal cycle of malaria prediction skill. Prediction accuracy is shown in function of target malaria prediction month (y-axis) and prediction lead (x-axis) for (left) exp1 which makes use of SST-based climate indices over the tropical Indian Ocean (IOD) and (right) SST over the western Pacific (WP; exp10). The skill is assessed with a multi-model voting ensemble composed of the three best classifiers selected for each lead/month combination. Accuracy is illustrated and indicated with numbers and colors as in Figure 5.

Prediction accuracies for the experiment using IOD-related predictors (exp1; Figure 6) are particularly high in December and January, which is a critical time for the planning of prevention interventions. For that purpose, predictions with accuracies around 80–90% can be provided up to ~4–6 months in advance. In contrast, accuracies for the predictions based on WP predictors are largest in austral fall, and peak at longer leads (5–8 months) with accuracies reaching up to 80–90%. In fact, WP indices provide by far the greatest accuracies in austral fall (Supplementary Figure 7). Overall, at long lead times, these accuracies are far superior to those of predictions based on malaria time series alone (Supplementary Figure 8).

We note that it may seem at first counterintuitive to see prediction accuracies increasing with time (instead of decreasing) in Figures 5, 6. This feature was supported by the lagged-correlation analysis shown in Figure 4. For instance, the correlation between malaria incidence and sst_WPnorth is largest at lags −7 to −8 for April malaria predictions and decreases for shorter lags. This indicates that the influence of SST variability over that sector on South African climate, and thus malaria, is most efficient several months before the outbreaks while weak just before the outbreak. This could be due to seasonal differences in the atmospheric teleconnection associated with sst_WPnorth variability, which may be sensitive to changes in the seasonal-mean flow. IOD-based precursors also exhibit a similar “seasonal locking” and this is ultimately reflected in prediction accuracy.

**Figure 7:**
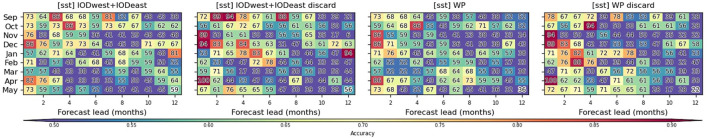
Predictions for Mopani: Similar to Figure 6 but for the district of Mopani instead of Vhembe. Results from additional experiments, labeled as discard, in which ambiguous categories and 2017 malaria data are discarded, are also shown.

Prediction accuracy for the district affected by the second-largest outbreaks, Mopani (Figure 1), is also tested in the study (Figure 7). In comparison to Vhembe, a general decrease of prediction accuracies is noted in December and January but it increases substantially at the beginning of the malaria season in September and October when using IOD-based climate predictors. A similar decrease in accuracy is observed when using the WP-based predictors.

Additional experiments are performed for Mopani by discarding malaria data for 2017, which is an outlier characterized by an extremely strong and unusual outbreak (above 4 standard deviations), as well as discarding samples between the 42.5 and 57.5 percentiles, whose classification into high and low incidence categories is more ambiguous and susceptible to measurement errors. By discarding these ambiguous samples, a non-negligible improvement of the accuracy is observed in December and January for IOD-based experiments and a similar increase is observed for these 2 months for WP-based experiments.

### Teleconnections

**Figure 8:**
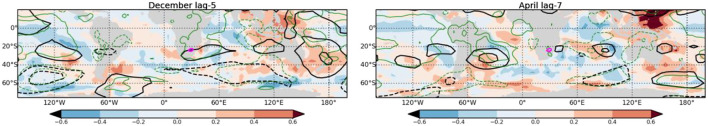
Atmospheric teleconnections: 500-hPa (black contours) and sea level pressure (green contours) precursor to December (left) and April (right) malaria outbreaks are shown for −5 months and −7 months lags, respectively. Precursors are illustrated as correlations with solid and dashed contours (intervals of 0.2) for positive and negative correlations respectively. They are overlaid on SST precursors shown with red/blue shading. Correlations above 0.4 and below −0.4 are significant at the 95% significance level.

Atmospheric circulation patterns preceding malaria outbreaks are briefly investigated to qualitatively verify the processes originating from slowly varying SST anomalies/indices in selected regions. They are shown for a 5-month lag preceding December malaria outbreaks and a seven-month lag preceding April malaria outbreaks, periods when IOD-related predictors and WP-related predictors contribute to higher prediction accuracies, respectively (Figure 8). We note that precursors of December malaria outbreaks are characterized by enhanced 500-hPa geopotential height and surface pressure in the vicinity of South Africa, indicating the presence of a barotropic high-pressure system. Circulation anomalies are also observed at remote locations, in alternating patterns of high and low anomalies, suggesting that the generation and propagation of quasi-stationary Rossby wave trains may play a role in producing the local response. Precursors of April outbreaks are less obvious in South Africa, with a weak hint of enhanced 500-hPa height and surface pressure. The detailed study of dynamical processes, such as the forcing by SST anomalies, leading to these responses, their impact on local weather, and factors that contribute to their impact on malaria outbreaks at such long lead times will be the topic of future work.

### Sensitivity to machine-learning setup

#### Classifier performance

Overall, it is found that linear discriminant analysis and logistic regression are the classifiers that offer the greatest accuracy for the peak malaria season (Supplementary Figure 9). We note that at short lead times, persistence is often among the most accurate classifiers suggesting that information about short-time scale climate information is not adding value to predictions. At longer lead times, however, the standard classifiers often exhibit higher accuracy, indicating the value of slowly varying climate information. An exception is for the month/lead combinations where accuracy is very low and for the beginning and end of the high-risk season when malaria persistence is highest (Figure 2). We also note that the multi-model voting ensembles are rarely the best but serve here as a more conservative indicator of prediction accuracy since the members are selected without knowledge of the prediction outcome, as is the case for real predictions.

#### Categories

As a standard, we use binary classification—more than usual, and less than usual—as described earlier. We stress that classification is performed month-by-month thus no seasonal cycle is present in the classification outcome, i.e., each month has an almost equal number of years classified as high and low incidence. Predictions with more classes: 3 classes (divided by the 33rd and 66th percentiles), 4 classes (divided by the 25th, 50th, and 75th percentiles), and 5 classes (divided by the 20th, 40th, 60th, and 80th percentiles), were also briefly investigated. Only the results of the 3-classes predictions are reported (expS2). Predictions with three classes show overall an important drop in accuracy (Supplementary Figure 10) but are above what is expected for random guesses (33.3%). Beyond 3 classes (not shown), accuracies are quite low and are likely of little value for decision-makers.

#### Lags included

For most predictions, only the last month of observations is provided (indicated in blue in Supplementary Figure 4). This is the most sensible choice to learn about the temporal origin of predictors. Using many time steps at once would blur this information. We nonetheless test the impact on predictions when including a long history of predictors (12 months in expS4). It is found that it overall does not improve the accuracy, likely due to overfitting as a result of the excessively large number of predictors compared to the samples available for training (Supplementary Figure 10).

#### Smoothing

We test the impact of smoothing by turning it off in one experiment (expS1). This results in a significant drop in accuracy, especially at long lead times (Supplementary Figure 8). This indicates that it is beneficial to exclude high-frequency weather noise, and focus on variability on the seasonal time scale and longer for skillful predictions at long lead times.

#### Trends

Although the identification of precursors through lag regression uses detrended data, actual predictions are performed with raw data because to plan interventions, it is useful to provide forecasts relative to the historical record. We show briefly, however, the impact of detrending in Supplementary Figure 11. For this exercise, we use exp1 for malaria prediction in December based on a 5-months lead time. Comparing raw to detrended malaria incidence, we observe a non-negligible impact on malaria classification and prediction. In raw data, a clear downward trend in malaria incidence is observed which results in a higher frequency of higher-than-usual incidences for the first half of the record compared to the second half. Detrended data, in contrast, shows a greater balance of classes throughout the record. This greater balance is however accompanied by a reduction in prediction accuracy (0.68 vs. 0.86). Overall a small reduction in accuracy is observed in austral summer (expS3 in Supplementary Figure 10).

#### Impact of sample size

As in all machine-learning applications, the quantity of data has a strong impact on prediction accuracy. Generally, the prediction skill increases as more recent data is made available (Supplementary Figure 12) and is expected to further improve over time as more malaria case data becomes available to train the machine learning models by reducing uncertainties in the climate-malaria connection, highlighting the importance of continuous and long-term monitoring of malaria in malaria-endemic countries. We note that discarding the first few years seems to improve the overall prediction accuracy, indicating that early malaria records may not be of the same quality as in more recent years.

## Summary and discussion

Early warning predictions of malaria outbreaks, by providing estimates of future infection rates with lead times of one to two seasons (3–6 months), are valuable to decision-makers for the effective planning of intervention measures. These measures, such as the distribution of mosquito nets, the indoor spraying of insecticides, and the supply of drugs to handle a rise in the number of patients are effective against the transmission and spread of malaria and greatly contribute to reducing malaria-related deaths and socioeconomic burden (1).

To assist this planning, and contribute to the fight against malaria by providing skillful predictions with sufficient lead time for the planning of intervention measures, a novel machine-learning-based approach to malaria prediction is developed based on remote SST variability, offering impressive prediction skills up to three seasons ahead (6–9 months). A unique feature of this prediction system is that it provides predictions of malaria incidences locally based on remote SST variability through computationally cost-effective machine learning methods. More specifically, a set of machine-learning classifiers is trained and optimized to predict whether malaria incidences rise above or fall below normal values.

Through the development of this prediction system for the province of Limpopo in South Africa, we identified a sector over the western Pacific Ocean from which past information about SST offers impressive prediction skills (~80% accuracy) up to three seasons (9 months) ahead (Figure 6). SST variability over the tropical Indian Ocean is also found to provide good skills up to two seasons (6 months) ahead. This outcome represents an extension of the effective prediction lead time by about one to two season compared to previous prediction systems (40, 41) that were more computationally costly, and less suitable for decision-makers in unindustrialized malaria-endemic countries.

In contrast to these SST-based sources of predictability, local weather information provides predictive power only at much shorter lead times (1–2 months), actually too short to be valuable to plan intervention measures. In addition, malaria-endemic countries traditionally relied on predictions based on local weather which lacked information on weather conditions in the neighboring countries which is important for overall malaria incidences through migration. This, together with the unavailability of inter-country migration numbers, make predictions difficult in those traditional methods. These issues are to a great extent covered by the large-scale climate phenomena investigated here that influence wider regions through teleconnection mechanisms.

While the actual mechanism(s) through which these climate phenomena and associated teleconnections affect the local weather remains to be understood, the prediction framework developed herein could be useful to predict malaria for other sectors of Africa, and potentially for the South-East Asian, Eastern Mediterranean, and American sectors also afflicted with malaria (1). These other sectors likely have distinct relationships between climate variability and regional malaria incidence that remain to be uncovered with the techniques developed herein. More extensive research on the connection between malaria and climate variability, including the search for new predictors, is expected to further contribute to our understanding of the climate's impact on malaria, improve malaria predictions and reduce the related socioeconomic burden through informed decision-making.

## Data availability statement

The data analyzed in this study is subject to the following licenses/restrictions. Due to the sensitive nature of the malaria case data, it may unfortunately not be publicly shared. Data are however available from the authors upon reasonable request and with permission of the National Department Of Health of South Africa. Only de-identified data can be made available. Requests to access these datasets should be directed to pmartineau@jamstec.go.jp.

## Author contributions

PM led and coordinated the various components of the study throughout and took the lead in writing the manuscript. PM, SB, MN, RJ, TI, NM, PK, and QM discussed the results, and aided in their interpretation. All authors contributed to the article and approved the submitted version.

## Funding

This research was carried out for the iDEWS project supported by SATREPS Program of JICA/AMED in Japan and ACCESS (NRF/DST) in South Africa.

## Conflict of interest

Author TI was employed by Blue Earth Security Co., Ltd. The remaining authors declare that the research was conducted in the absence of any commercial or financial relationships that could be construed as a potential conflict of interest.

## Publisher's note

All claims expressed in this article are solely those of the authors and do not necessarily represent those of their affiliated organizations, or those of the publisher, the editors and the reviewers. Any product that may be evaluated in this article, or claim that may be made by its manufacturer, is not guaranteed or endorsed by the publisher.
