# Supplementary material for: Predicting malaria outbreaks from sea surface temperature variability up to 9 months ahead in Limpopo, South Africa, using machine learning
Source: Front Public Health. 2022 Aug 25;10:962377. doi: 10.3389/fpubh.2022.962377 (PMC9453600; doi:10.3389/fpubh.2022.962377)
Supplement: Supplementary file 1 [file Data_Sheet_1.docx]

Supplementary Material

**Supplementary Table 1: List of machine-learning classifiers used for malaria incidence predictions.** The machine-learning classifiers trained and used to predict malaria incidence are listed with their corresponding Python module. It is indicated whether these classifiers are based on ensembles of base estimators. Multimodel classifiers, 3 best and 5 best, are constructed by averaging the votes of the 3 and 5 best classifiers. The choice is made independently for each prediction month and lead time. The persistence classifier is a custom classifier that assumes malaria incidence does not change over time. It does not make use of climatic precursors.

**Supplementary Table 2: List of supplementary experimental setups.** The details of each experimental setup are indicated.

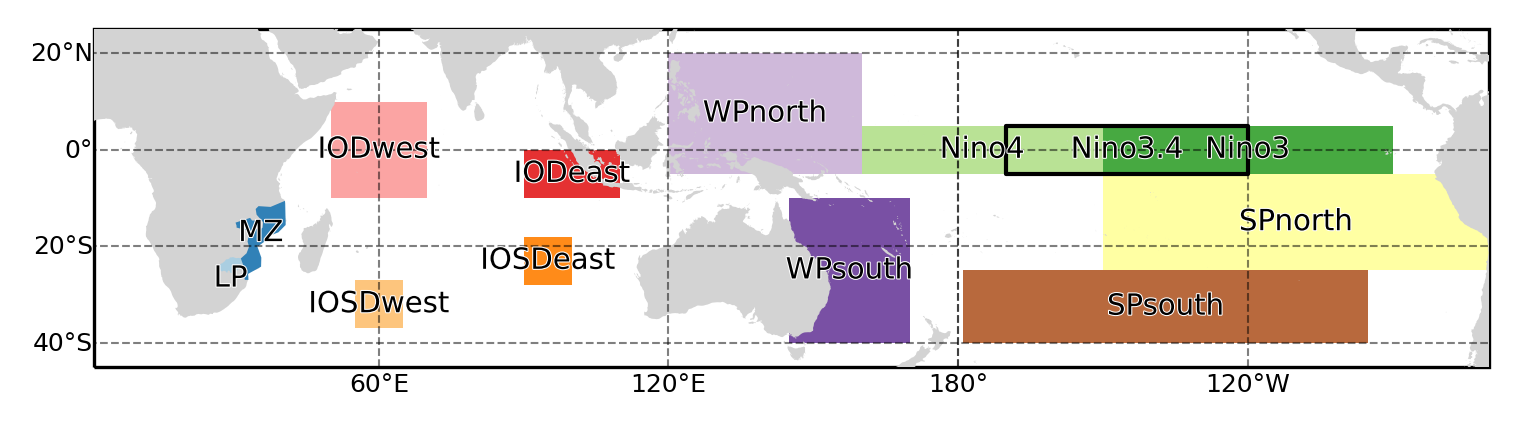


**Supplementary Figure 1: Domains used to construct SST-based climate indices and local climate indices.** The boundaries of SST-based climate indices are listed in Table 2. LP and MZ stand for Limpopo and Mozambique, respectively.

**
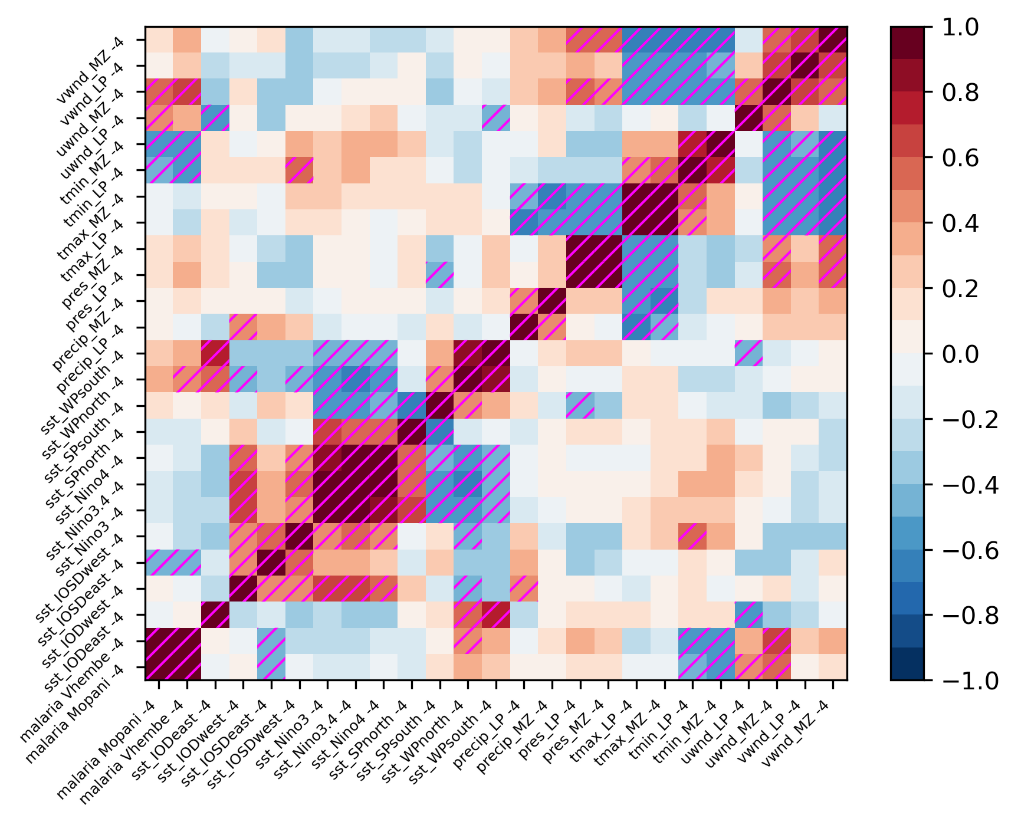
**

**Supplementary Figure 2: Cross-correlations between predictors.** February predictors of malaria are correlated amongst each other at lag -4 (thus observed in October). This lag/month combination is chosen to illustrate that cross-correlations are found among predictors. Other lags and months also show significant cross-correlation among indices (not shown). Correlations that are significant at the 5% significance level are hatched in magenta.

**
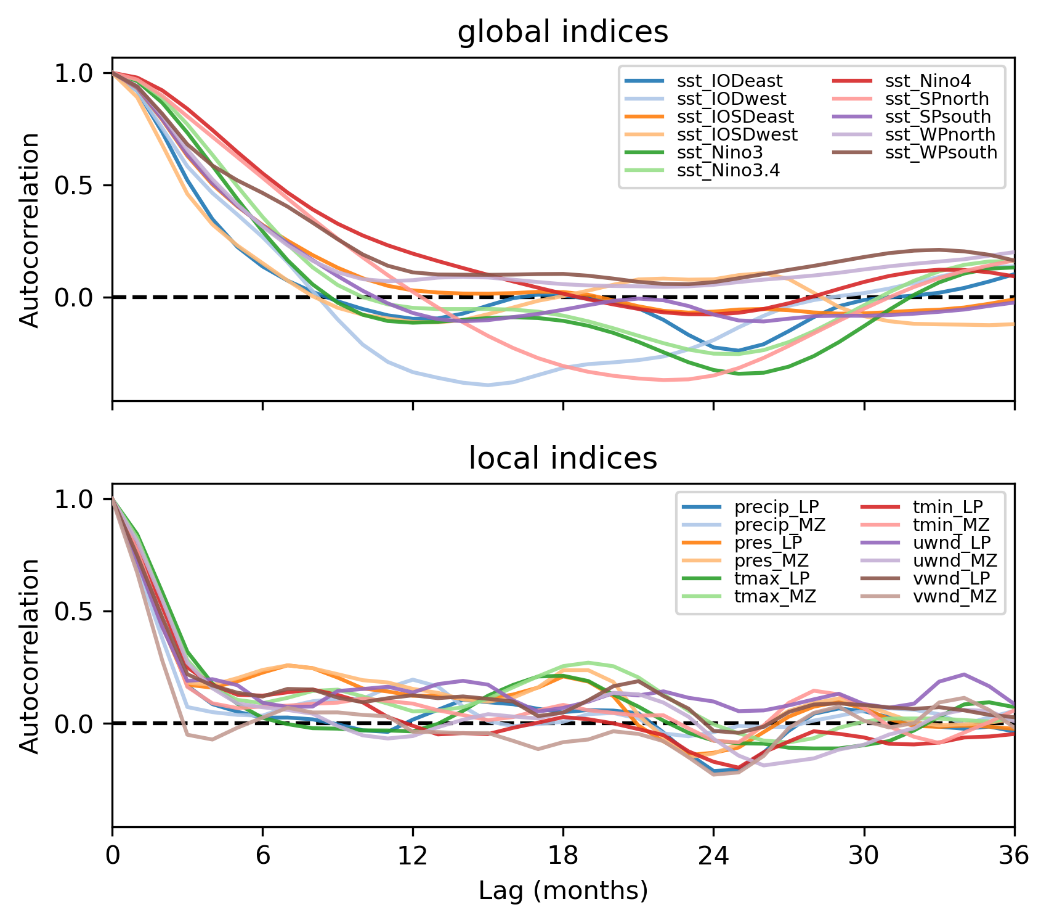
**

**Supplementary Figure 3: Autocorrelation of climate indices.** Global (top) and local (bottom) climate indices (color-coded according to the legend) are correlated with lagged replicas of themselves to assess their time scale of variability. Note that the original time series are subjected to a 3-month running mean before the correlation analysis is performed. Most local indices, except tmax and tmin, have faster decorrelation times in comparison to global SST-based indices.

**Supplementary Figure 4**: Diagram illustrating the predictors used for each prediction. An x-month prediction is defined as a prediction based on predictors x months before the prediction date. Unless specified, only one month of predictors (climate indices & malaria; blue) are provided. Prediction accuracy is assessed for predictions based on a 12-month climatic history (red/blue; malaria provided only for the last month) in expS3.

**Supplementary Figure 5: Model training and skill evaluation.** The machine-learning classifiers are trained and assessed with nested cross-validation. In the outer loop, classifiers are trained on the outer training and their skill is evaluated on the test data. For each iteration of the outer training an inner loop is performed, where hyperparameter tuning is carried out by training the classifiers on the inner training and assessing their skill on the validation data.


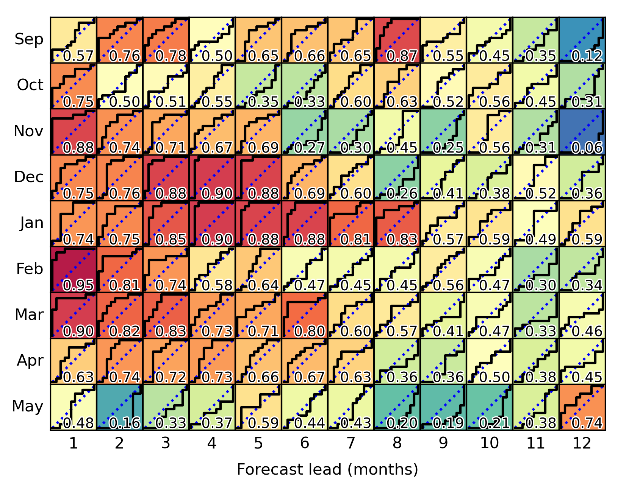

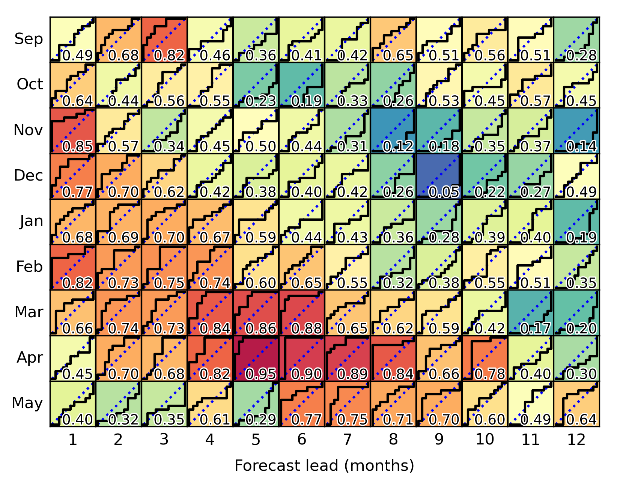


**Supplementary Figure 6: Receiver operating characteristic (ROC) curves.** ROC curves (black lines) are illustrated for exp1 (SST_IODwest+IODeast_, left) and exp10 (SST_WP_, right) for each month (y-axis) and lead time (x-axis). For each subpanel, the y-axis shows the true positive rate and the x-axis shows the false positive rate, both ranging from 0 to 1. The curve corresponding to random classification is shown with dashed blue lines. The area under the curve (AUC), an aggregate measure of performance across all possible classification thresholds (ranging from 0-1), is indicated in each panel. The AUC is also illustrated as the background color of each subpanel with warmer colors indicating higher AUC.

**
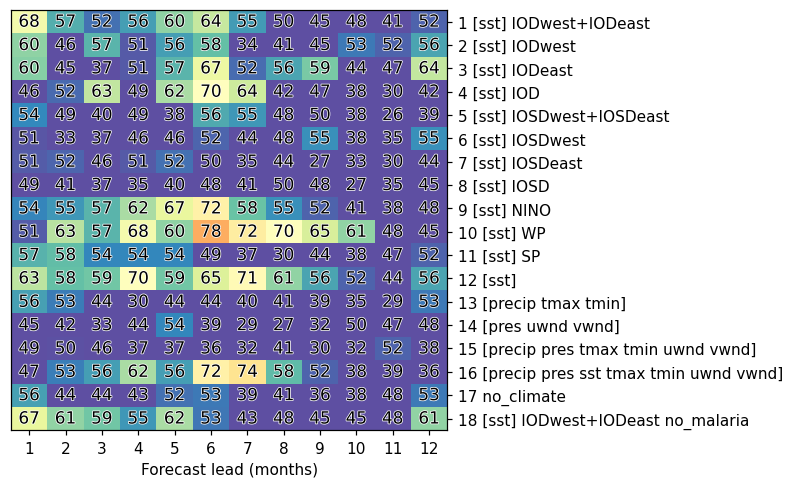
**

**Supplementary Figure 7**: Prediction accuracies in austral fall. Same as Fig. 5 except accuracies are reported for Mar-May.


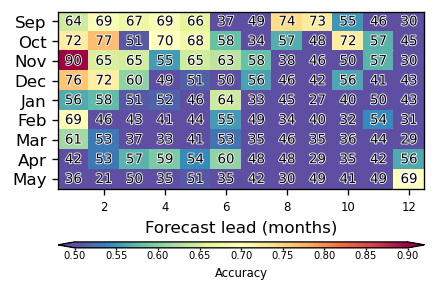


**Supplementary Figure 8**: Same as Fig. 6 except for exp 17 which only uses malaria data to make predictions.

**
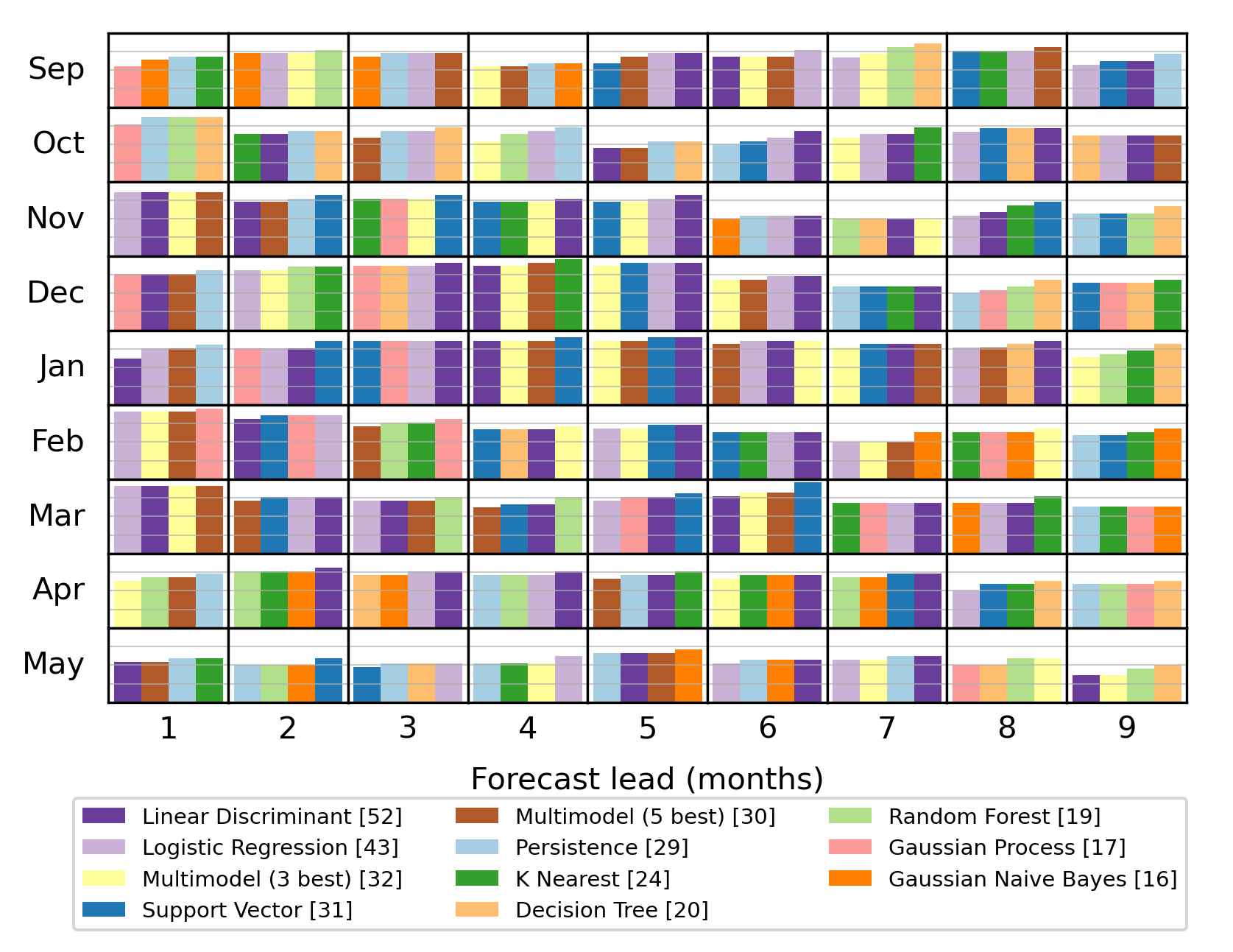
**

**Supplementary Figure 9: Classifier performance.** For each month of the year (y-axis) and each lead time (x-axis), the performances of the four best classifiers are illustrated with bars for exp1. The maximum bar height represents an accuracy of 100% (or 1). Colors indicate the type of machine-learning classifier as indicated in the legend. The total number of times that a classifier is counted among the four best is indicated in square brackets in the legend.


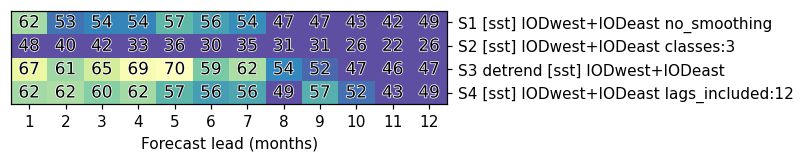


**Supplementary Figure 10: Assessment of machine-learning techniques.** Same as Fig. 5, but for the experiments listed in Supplementary Table 2.

**
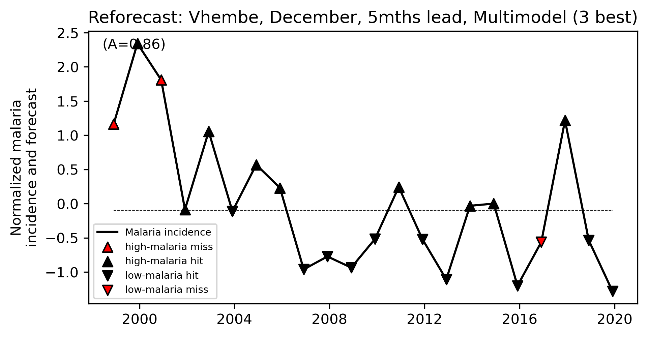

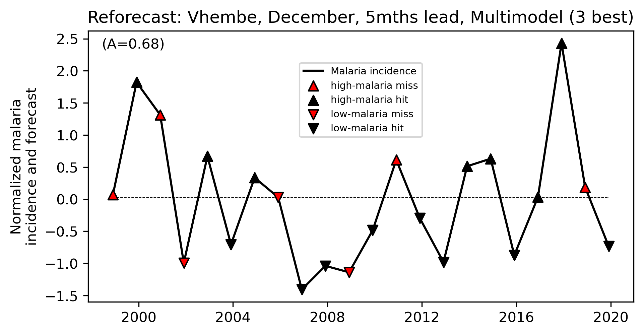
**

**Supplementary Figure 11: Impact of trends:** The December normalized malaria incidence, its classification into high and low-incidence categories, and 5-months lead forecast results (hit or miss) are shown for experiments using absolute (left) and detrended (right) malaria incidences. The accuracy is indicated in parentheses on each panel. The median is shown as a dashed line.

**
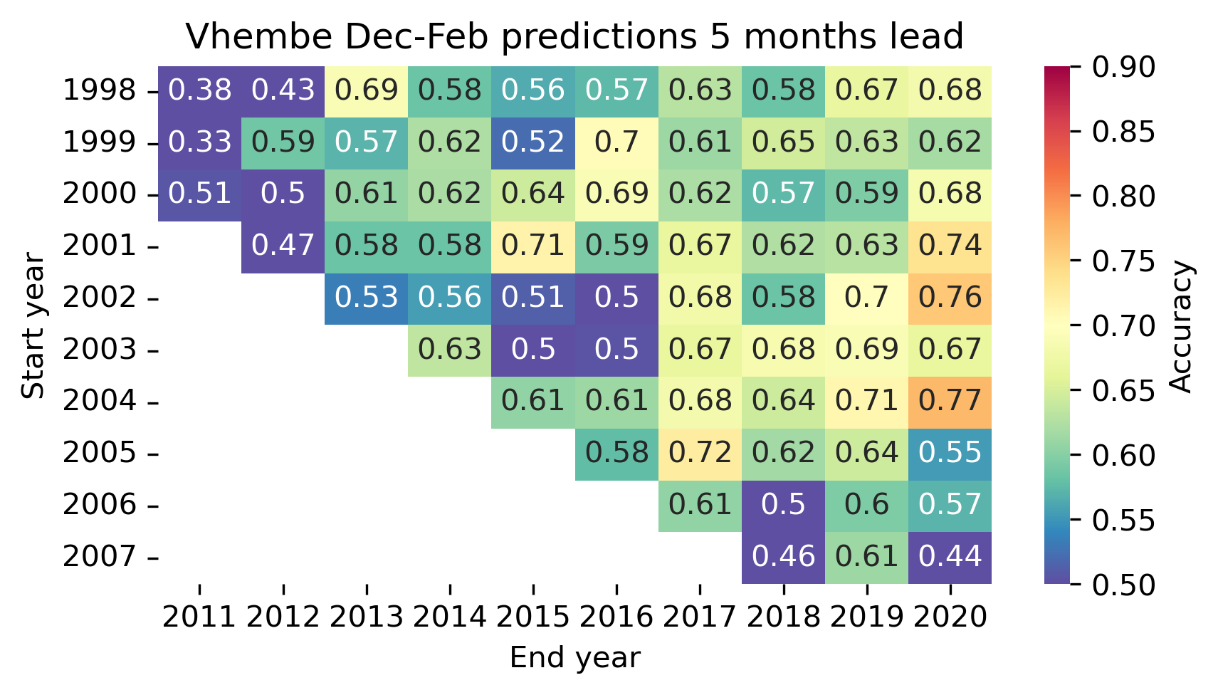
**

**Supplementary Figure 12: Influence of sample size on prediction accuracy.** The influence of sample size on austral summer (December-January-February) prediction accuracy for exp1 with a five-month lead time is assessed by systematically altering the start year (y-axis) and end year (x-axis) of historical malaria data.
